# Electronic transport properties of PbSi Schottky-clamped transistors with a surrounding metal–insulator gate

**DOI:** 10.1039/c7ra11653e

**Published:** 2018-01-04

**Authors:** Lishu Zhang, Yifan Li, Tao Li, Hui Li

**Affiliations:** Key Laboratory for Liquid-Solid Structural Evolution and Processing of Materials, Ministry of Education, Shandong University Jinan 250061 People's Republic of China lihuilmy@hotmail.com

## Abstract

Sustaining Moore's law requires the design of new materials and the construction of FET. Herein, we investigated theoretically the electronic transport properties of PbSi nanowire Schottky-clamped transistors with a surrounding metal–insulator gate by employing MD simulations and the NEGF method within the extended Hückel frame. The conductance of PbSi nanowire transistors shows ballistic and symmetrical features because of the Schottky contact and the resonance transmission peak, which is gate-controlled. Interestingly, the PbSi(8,17) nanowire FET shows a high ON/OFF ratio and proves to be a typical Schottky contact between atoms as described by the EDD and EDP metrics.

## Abstract 2

Sustaining Moore's law requires the design of new materials and the construction of FET.

## Graphical abstract

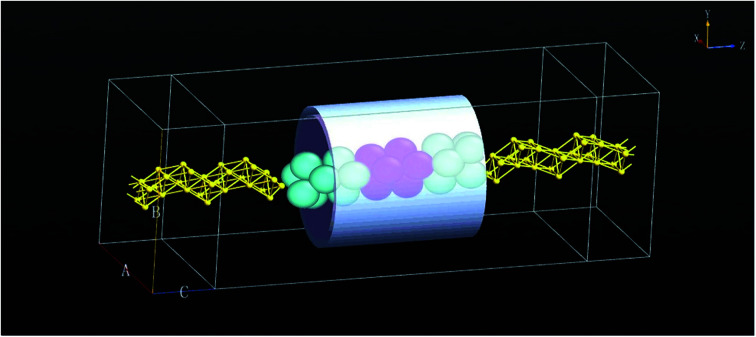

## 1. Introduction

The reduction in dimensions of electronic devices and the introduction of additional performance boosters such as metal gate electrodes and high-k gate dielectrics have proved to be a remarkable roadmap for improving transistor performance in the last decade.^2–4^ In addition, the integrated circuit (IC) industry has taken advantage of low-dimensional materials to manufacture gate-control transistors. For example, Yang *et al.*^5^ developed the graphene barristor, a triode device with a gate-controlled Schottky barrier, by adjusting the gate voltage to modulate the device current and achieve a high ON/OFF ratio of 105. In addition, Jan *et al.*^6^ optimized a leading edge 22 nm-3-D tri-gate transistor technology for low power SoC (system on chip) products for the first time. Although the technology for tri-gate transistors in low dimensions has undergone huge developments, reducing device size and diminishing device dimensions remain a challenge for current technology worldwide.

Nanowires, which are one-dimensional materials, have gained tremendous interest as novel materials for next-generation electronic devices because they successfully address the formidable challenges of transistor scaling. Recently, Rim *et al.*^7^ fabricated phosphorus-doped silicon nanowire field-effect transistor biosensors using conventional CMOS techniques; their low frequency characteristics are measured by the noise equivalent gate voltage fluctuation and exhibit drastically enhanced performance. Furthermore, Zheng *et al.*^8^ successfully produced n-type silicon nanowires (SiNWs) *via* a controlled phosphorus dopant technique for the first time and prepared high-performance n-type FETs from these n-SiNWs, which exhibit excellent device properties with mobilities more than 100 times larger than previously observed results and comparable to the present silicon FETs. All these findings provide a new means for reversibly changing the electronic properties of nanowire electronics; however, they are not adequate to meet the increasing demand of new materials^1^ in the field of electronic devices. Previous studies^9^ indicated that lead nanowires doped with silicon exhibit ballistic conductance features and a negative differential resistance effect, confirming that Pb–Si nanowires hold promise for use in molecular devices, quantum dot devices, and silicon-based field-effect transistors. However, sufficient studies based on PbSi nanowire devices have not yet been conducted. Herein, we studied a type of metal–insulator–PbSi nanowire FET and its potential prospects for the development of next-generation devices.

## 2. Models and computational methods

To obtain stable nanowire structures, the Forcite module of MATERIALS STUDIO was employed to perform energy minimizations of different sets of structures by including all the atomic degrees of freedom.^10,11^ First, we randomly inserted Si and Pb atoms into single-wall carbon nanotubes. Then, we performed geometry optimizations to obtain stable configurations. The iterative process was not performed before the number of the maximum iterations was 10^5^. To model the interatomic interactions in the optimization process, the universal force field was used. The parameters were generated from a set of rules according to elements, connectivity and hybridization. Moreover, we parameterized the universal force field for the full periodic table. We set the energy convergence tolerance as 0.001 kcal mol^−1^ and the force convergence tolerance as 0.5 kcal mol^−1^ Å^−1^ to enhance the computational accuracy. The atomic coordinates were adjusted before the total energy of the structure attained a minimum.

The current–voltage characteristics and the electron transmission probability of the optimized nanowires were calculated using the method reported by Datta *et al.*^12,13^ To resolve convergence issues and improve calculation speed, the extended Hückel theory (EHT) and nonequilibrium Green's function formalism (NEGF) were employed. This approach ensured sufficient precision.

**Fig. 1:**
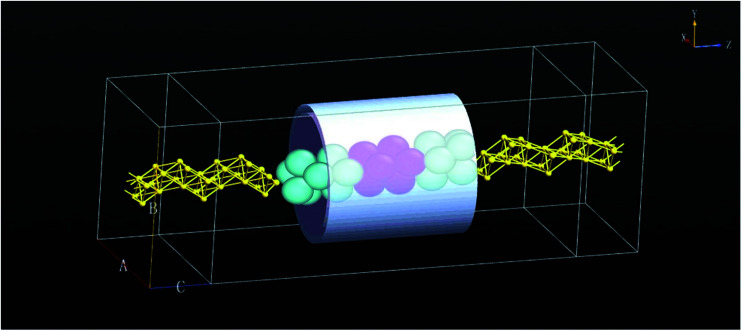
Schematic of the PbSi nanowire FET surrounded by a metal–insulator gate (the dielectric constant and the thickness of dielectric layers are 3.9*ε*_0_ and 3 Å). The electronic device is composed of three sections that are from left to right: the source, scattering and drain regions. The grey tube is the gate electrode, and the Au atoms are represented in gold.

The PbSi nanowire is chemically bound to two Au(111) electrodes, each of which have two layers of surface atoms on each side to interact with the device. In this study, we added a tube gate electrode with dielectric layers consisting of an oxide as shown in Fig. 1. This computational model is composed of two well-defined independent sections: one is the “scattering region” and the other is the “contacts”.^14^ The scattering region is represented by a Hamiltonian matrix, which is divided into two parts: (i) one is the self-consistent part, *H*_SC_, which denotes the charging and the screening effect in the device and can be affected by the source-drain voltage and the gate voltage; (ii) the other is a core Hamiltonian matrix, *H*_0_, which is obtained from EHT.

Most of the electronic transport properties of the nanowire are derived from the nonequilibrium Green function formalism:

1 *G*(*E*) = (*ES* − *H* − *Σ*_1_ − *Σ*_2_)^−1^

The self-energy matrices *Σ*_1,2_, which are used to describe the effect of the semi-infinite Au(111) contacts on the nanowires, are calculated by a recursive technique.^15^ The electron density is calculated by the following formula:2
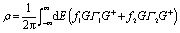
*G* and *G*^+^ denote the Green's function matrices reflected from the left electrode and the right electrode to the central scattering region.

The equation for the current, *I*, through the device is based on eqn (1) and (2) as follows:3
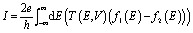


The quantity *T*(*E*,*V*) is the transmission function, which expresses the transport probability of electrons transmitting through the device from the source to the drain; *f*_1,2_(*E*) denote the Fermi functions of the source and drain electrodes; *e* and *h* are the electron charge and Planck constant, respectively.

The transmission *T* represents the electron transmission probability:

4 *T*(*E*,*V*) = trace(*Γ*_1_*GΓ*_2_*G*^+^)

## 3. Results and discussion

**Fig. 2:**
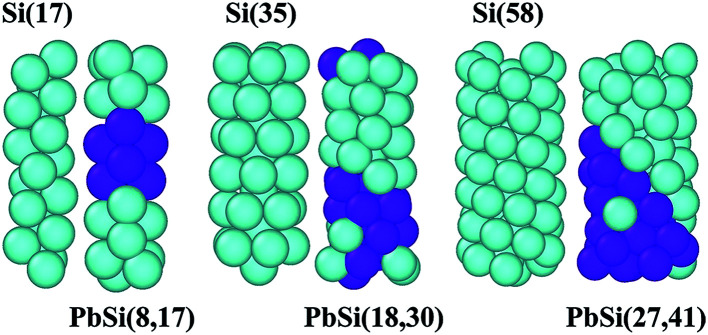
The structures of pure Si(17), Si(35) and Si(58) nanowires and PbSi(8,17), PbSi(18,30) and PbSi(27,41) nanowires, respectively. Si atoms are represented in blue, and Pb atoms are represented in violet.

The optimized configurations are shown in Fig. 2, in which Si atoms are represented in blue and Pb atoms in violet. We named these PbSi nanowires according to the respective number of Pb and Si atoms in the nanowires: PbSi(8,17), PbSi(18,30) and PbSi(27,41). It is evident that all PbSi nanowires have structures similar to that of pure Si nanowires as they were obtained from the same CNT. PbSi(8,17), PbSi(18,30) and PbSi(27,41) were obtained from (8,8), (10,10) and (12,12) CNTs, respectively. As observed, the diameter of the nanowire influences its structure. The Si(17) and PbSi(8,17) nanowires obtained from the (8,8) CNT are formed by two-strand helical chains, while the Si(35) and PbSi(18,30) nanowires obtained from (10,10) CNT are composed of five-strand helical chains with a one-strand atomic chain core. The nanowires inside the (12,12) CNT consist of a shell formed by eight-strand helical chains and a one-strand atomic chain core. Our as-prepared nanowire structures are in agreement with previously reported structures^10,16–19^ using the same method, which illustrates the reasonability of our results. However, the main outcome is that all alloy nanowires exhibit a segregation phenomenon, for example, the PbSi(8,17) nanowire appears as a regular Si–Pb–Si structure, and it can be regarded as two Schottky contacts. This separation may be due to different types of atoms having different interactions. During the structure optimization using MD method, the Pb atoms are found to condense and coalesce more easily than the Si atoms due to stronger Pb–Pb and Si–Si interactions, relatively weak Pb–Si interactions and a barrier between two adatoms.^20,21^ The doping concentrations of lead in the PbSi devices are 29.17%, 40.43% and 40.30% for PbSi(8,17), PbSi(18,30) and PbSi(27,47), respectively, indicating that the concentration of lead in (8,17) PbSi device is the lowest, while those in (18,30) and (27,41) PbSi devices are high. When the doping concentration is low, the heat emission current in the junction would play a major role, and the contact resistance depends on the barrier height rather than the doping concentration. However, the tunneling effect primarily affects highly-doped metal–semiconductor junctions. Therefore, the three doped PbSi nanowire devices only form a metal (Pb)–semiconductor (Si) Schottky contact that would produce a current through the metal and the semiconductor side.

**Fig. 3:**
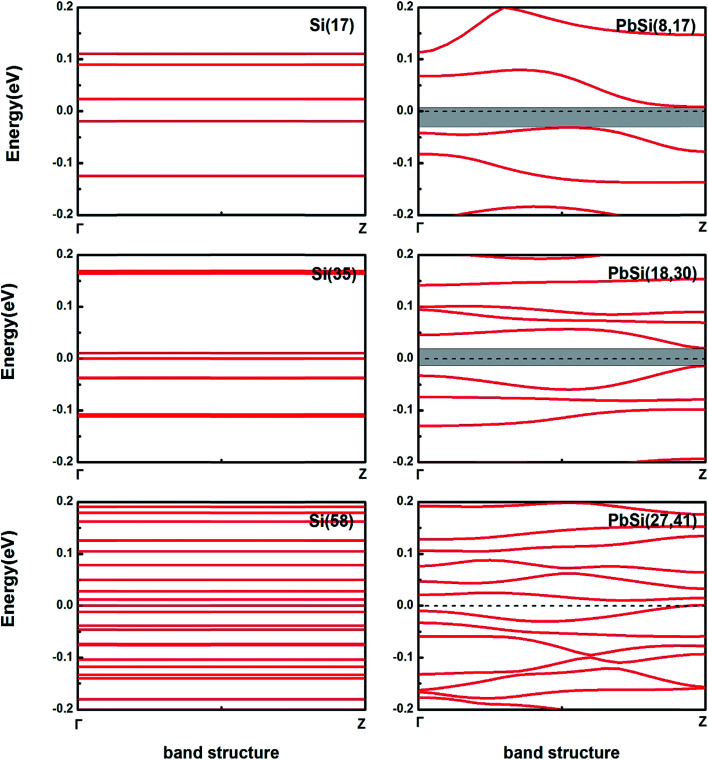
Band structure of Si and PbSi nanowire devices. The dashed line represents the Fermi level, which is set as 0 eV. The shadow window indicates bandgap. The PbSi(27,41) nanowire device has a band in the Fermi level.

In order to identify the semiconductor characteristics of the alloy nanowires, we calculated the band structure of the PbSi(8,17), PbSi(18,30) and PbSi(27,41) nanowire devices as shown in Fig. 3. All three PbSi nanowire devices have indirect bandgap semiconductor properties since their electronic distribution in *k*-space varies during the electronic transition process. The bandgap of the PbSi nanowires becomes narrower with an increase in diameter due to quantum confinement effects and the Coulomb blockade effect. The PbSi(8,17) and PbSi(18,30) devices show semiconductor characteristics, while PbSi(27,41) exhibits semimetal feature because of the presence of only one band in the Fermi level, which results from not only the doping concentration, but also from the distribution of the doping atoms. What counts is that the band structures of the PbSi nanowires upsweep because the surface state density is high and the surface accumulates a large amount of negative charges.

**Fig. 4:**
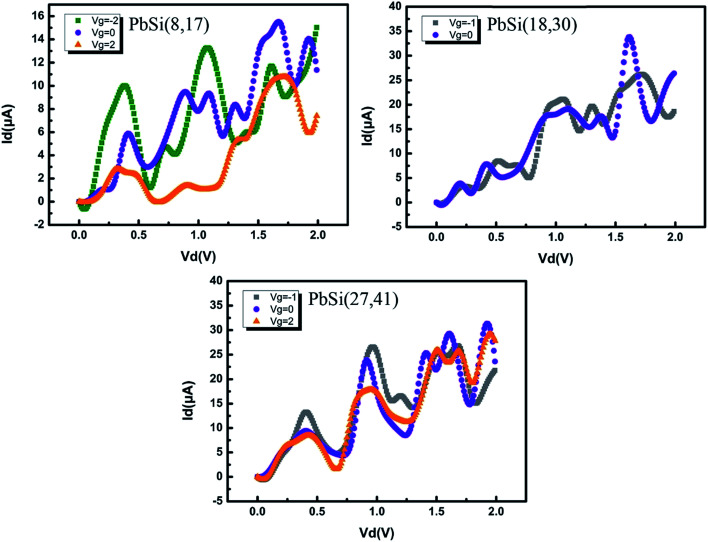
*I*–*V* curves of the PbSi(8,17), PbSi(18,30) and PbSi(27,41) devices at different gate voltages. The gate voltage corresponds to that of the transmission spectra.

To study the electrical properties of the PbSi devices, we further calculated the *I*_d_–*V*_d_ curves with different gate potentials as shown in Fig. 4. Compared to PbSi(18,30) and PbSi(27,41), the PbSi(8,17) device exhibits a drastic cut-off state and produces a reverse current when *V*_g_ = 2 V. Interestingly, the PbSi(8,17) device has a regular Si–Pb–Si distribution, and current flows from the left to the right electrode. In general, the Fermi level of pure silicon is higher than that of pure lead, while electrons in semiconductors flow to metals with lower energy level in order to allow the Fermi level to change continuously during thermal equilibrium. This leads to the formation of a space charge area (depletion layer) because positively charged holes remain in the semiconductor. In addition, this depletion layer, which is affected by the material, temperature and bias voltage, is unique to PN junctions. Moreover, we can conclude that all devices follow the general law: *I*_negative_ > *I*_equilibrium_ > *I*_positive_. Furthermore, we calculated the ON/OFF ratio of these devices; in addition, the current at negative *V*_g_, *I*_on_, and the off-state current at positive *V*_g_, *I*_off_, were obtained. The ON/OFF ratio is about 238 for PbSi(8,8), which exceeds the typical ON/OFF ratio for traditional semiconductor devices. If we follow the conventional definition to determine the ON and OFF voltage, the ON-state voltage *V*_on_ would have a value beyond the power supply voltage.^22,23^

**Fig. 5:**
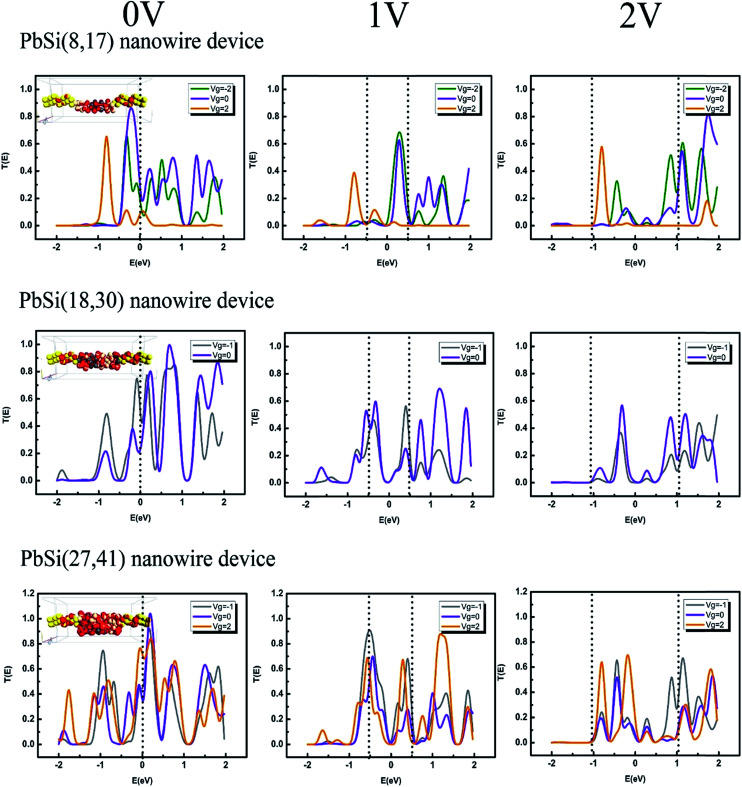
Transmission spectra of the PbSi(8,17), PbSi(18,30) and PbSi(27,41) devices at different states. The doted line represents the bias window. The inset is the LDOS at equilibrium.

To probe the origin of the *I*–*V* characteristics of the devices, the transmission spectra of these three PbSi nanowire devices at different bias voltages and gate voltages within the bias window are shown in Fig. 5. At zero bias, electrons can transfer through atomic junctions *via* resonant tunneling. Upon applying a certain bias, the position of the occupied and unoccupied molecular orbitals aligns with the Fermi level and therefore, allows for electron transport through atomic junctions on using these orbitals as the dominant transport channel. Orbitals with energy close to the Fermi level will contribute to the current and show peaks near the Fermi energy in the bias window of the transmission spectra. These transmission spectra indicate that the more enhanced the peaks within the bias window at the same *V*_d_, the stronger the current exhibited. The number of peaks and their intensities increase when the diameter of the nanowires increases, which also results in stronger currents. As inferred from the transmission spectra under the same applied biases, the gate potential can have an effect on the peaks. When |*V*_g_| ≠ 0, the peaks within the bias window are enhanced, which weakens the conductance as shown in Fig. 5(c). The inset presents the corresponding LDOS, showing the strong locality between the Schottky contacts.

**Fig. 6:**
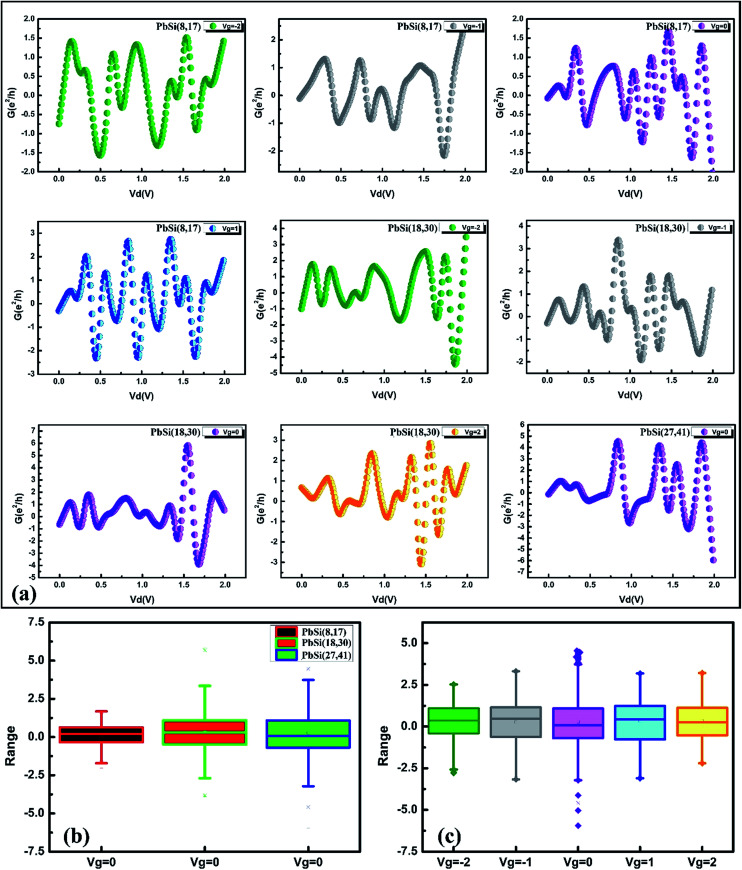
(a) Conductance *versus V*_d_ for PbSi devices at different gate voltages. (b) Oscillation amplitude range for the PbSi(8,17), PbSi(18,30) and PbSi(27,41) devices at equilibrium. (c) Oscillation amplitude range for the PbSi(27,41) device at different gate voltages. The grey line represents the trend in ranges with gate voltage.

We further determined the conductance of these three PbSi devices as shown in Fig. 6. It can be observed that all the devices exhibit dampened oscillations at different gate voltages, indicating ballistic electronic transport, which is in agreement with previous results,^9^ illustrating that the oscillation does not disappear under the gate voltage. In addition, most of the inflection points of the conductance curves appear at an integral multiple of the basic conductance (the basic conductance is *e*^2^/*h*). This phenomenon is in agreement with the experimental results for conductance in gate electrode structures.^24^Fig. 6(b) shows the oscillation amplitude range of the PbSi(8,17), PbSi(18,30) and PbSi(27,41) devices at equilibrium. It can be observed that the amplitude of the oscillation increases as the diameter increases, implying an enhanced oscillation effect. This oscillation effect denotes a universal conductance fluctuation (UCF), whose order of magnitude is *e*^2^/*h* = 4 × 10^−5^ S, indicating that the amplitude of all the devices in the normal conducting zone (*λ*_F_ ≪ l ≪ *L* ≪ *L*_φ_) is significantly dependent on the device diameter. Fig. 6(c) presents the oscillation amplitude range of the PbSi(27,41) device at different gate voltage, which follows the relation—the smaller |*V*_g_| has a larger range. Interestingly, when the gate voltage is zero, a conductance fluctuation phenomenon still occurs. When the bias voltage is greater than the gap between the one-dimensional sub-bands, the number of sub-bands between the forward and the backward transport is not equal, thus causing nonlinear conductance, which further leads to the negative differential resistance (NDR) of the *I*–*V* curve. Interestingly, we can clearly observe that the conductance of the PbSi(8,17) device exhibits a central symmetry at *V*_d_ = 1 V under a gate voltage of 1 V.

**Fig. 7:**
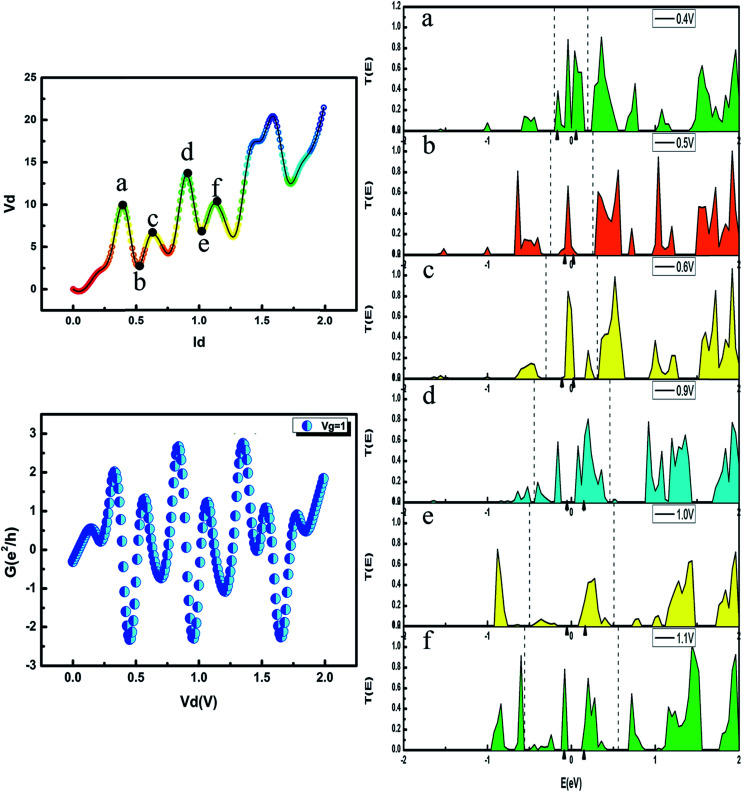
*I*_d_–*V*_d_ curve of the PbSi(8,17) nanowire device at *V*_g_ = 1 V and corresponding conductance curve. (a–f) Transmission spectra of the PbSi(8,17) nanowire device under a bias of 0.4, 0.5, 0.6, 0.9, 1.0 and 1.1 V, respectively. The dashed line represents the bias window. The transmission peaks within the bias window mainly contribute to the current. The energy level of these transmission peaks is consistent with the molecular orbitals. The small black triangles in (a)–(f) indicate the molecular projected self-consistent Hamiltonian (MPSH) near the Fermi level. The Fermi level is set to zero.

To explore the origin of this symmetrical conductance, the corresponding *I*–*V* curve and transmission spectra at six typical biases are shown in Fig. 7. The energy interval of the chemical potential from the left to the right electrode is represented by the bias window between the dashed line. In general, the integrated transmission in the bias window positively correlates with the voltage. Due to the symmetrical conductance, the *I*–*V* curve between 0.25 V and 1.25 V is nearly the same; hence, we selected six typical points to analyze the apparent symmetry of the conductance. We define point a as corresponding to point d, point b corresponding to point e and point c corresponding to point f. Clearly, points a, c, d, and f are turning points of crests, while points b, and e are the two lowest points of troughs. Moreover, the two correlating points have similar peaks within the bias window. We can observe that the transmission spectrum of the maximum voltage value (point d) presents the maximum integral area. Similarly, points b and e are valleys and both have only one peak within the bias window. The observed transmission spectra are a direct consequence of the two closely correlating *I*–*V* curves, which in turn are responsible for the symmetry of the conductance. We also calculated the molecular projected self-consistent Hamiltonian (MPSH) of the PbSi(8,17) device at *V*_g_ = 1 V because there is some level of association with the transmission spectra. Two MPSH values around the Fermi level, indicated by small black triangles in Fig. 7(a–f), trigger the transmission peaks within the bias window due to the effect of the Frontier molecular orbitals. Because of the electrodes, the two molecular orbitals are broadened, resulting in almost corresponding broad transmission peaks within the bias window.^25^ In addition, the localized charge at the contact points create Schottky-like barriers that also exist in Pb–Si contacts. The heights of these barriers are distributed in the value of the negative bias voltage. Therefore, most of the transmission in the negative bias is blocked except for two broadened molecular orbitals near the Fermi level.

**Fig. 8:**
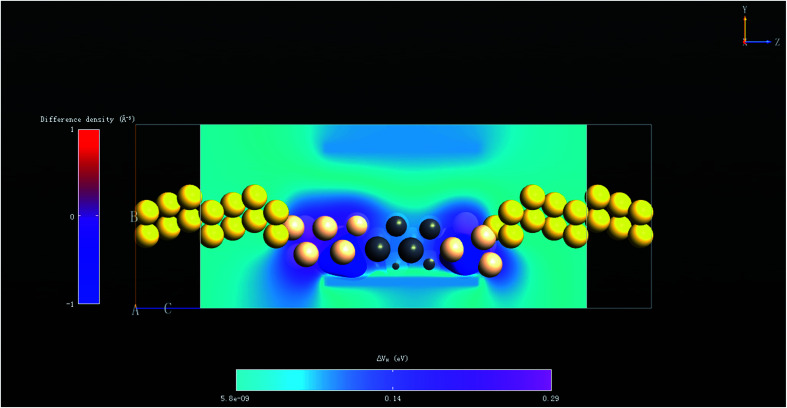
Electron difference density and electrostatic difference potential of the PbSi(8,17) nanowire device at *V*_g_ = −2 V and *V*_d_ = 1 V when the ON/OFF ratio is maximum. The EDP is shown through cut plane graphs with individual color bars. A potential is seen at the interface that connects the Pb metal potential to the Si semiconductor potential. Abscissa axis represents the electrostatic difference potential in eV. The vertical axis represents the distance along the *C*-direction. EDD shows isosurface graphs with individual color bars. Abscissa axis represents the electron difference density in Å^−3^. Vertical axis represents the distance along the *C*-direction.

To shed light on the link between the Schottky contact and the high electronic performance of the PbSi(8,17) nanowire device, we further calculated its EDD (electron difference density) and EDP (electrostatic difference potential) at *V*_g_ = −2 V and *V*_d_ = 1 V when the ON/OFF ratio is maximum. These parameters are the metrics that can effectively depict the electronic interaction at particular positions. A deviation between an assumed standard or model electron density and the actual observed or DFT-computed electron density is depicted by EDD. In fact, EDD is the difference between the self-consistent valence charge density and the superposition of atomic valence densities.^26^ An interaction between the metal–semiconductor atomic surfaces implies that there is a non-negligible change in the electron density after the self-consistent simulation at their boundary. Moreover, high interactions produce enough electron density, which are indicated by the small values of EDD, when subtracted from the initial or neutral electron density and *vice versa*. As illustrated in Fig. 8, the difference in electron density is shown by a red-blue map, and the Si atoms that connect to the Pb atoms have the smallest difference (−1 Å^−3^), showing a maximum charge rearrangement. Thus, the maximum charge interaction occurs between Si and Pb and illustrates the maximum covalence between this type of metal and semiconductor atoms. The EDP represents the difference between the electrostatic potential of the self-consistent valence charge density and the electrostatic potential derived from atomic valence overlapping.^26^ The former electrostatic potential is obtained by inserting the self-consistent valence charge density into the Poisson equation. The 3D cut plane geometries are represented in a cool-map, which shows that there is a high potential for charge carriers at the Pb–Si interface and a barrier for Si, once again indicating that there is more covalent bonding for Si atoms than Pb atoms.

## 4. Conclusions

MD simulations and the NEGF method within the extended Hückel frame were performed to study the electronic transport properties of PbSi nanowires that can be regarded as Schottky-clamped transistors. The conductance of the PbSi device displays ballistic and symmetrical features and the *I*–*V* curves show a negative differential resistance effect, which can be explained through Schottky contacts. The total charge density was used to evaluate EDP and the difference between the self-consistent valence charge density and the superposition of atomic valence densities was depicted by EDD. The electron difference density and electrostatic difference potential of the PbSi(8,17) nanowire device show that there is a high potential for charge carriers at the Pb–Si interface, indicating that there is more covalent bonding for Si atoms than Pb atoms. This study provides theoretical evidence that Pb–Si nanowire transistors surrounded by a metal–insulator gate hold promise for use in molecular devices and silicon-based field-effect transistors.

## Conflicts of interest

There are no conflicts to declare.

## Supplementary Material
